# *N*-Cyclo­hexyl-*N*-{[3-(4,6-dimeth­oxy­pyrimidin-2-yl­oxy)pyridin-2-yl]meth­yl}4,6-dimeth­oxy­pyrimidin-2-amine

**DOI:** 10.1107/S1600536812013104

**Published:** 2012-03-31

**Authors:** De-Cai Wang, Yu-Jing Wang, Jun-Song Song, Ping Wei, Ping-Kai Ou-yang

**Affiliations:** a State Key Laboratory of Materials-Oriented Chemical Engineering, School of Pharmaceutical Sciences, Nanjing University of Technology, Xinmofan Road No. 5 Nanjing, Nanjing 210009, People’s Republic of China

## Abstract

In the title compound, C_24_H_30_N_6_O_5_, the cyclo­hexyl ring adopts a chair conformation, while the remainder of the mol­ecule adopts a U-shape. The dihedral angles between the pyridine ring and the pendant pyrimidine rings are 69.04 (12) and 75.99 (9)°. The two pyrimidine rings, however, are nearly parallel to one another, with a dihedral angle of 8.56 (15)° between them. They are also involved in an intra­molecular π–π stacking inter­action with a distance of 3.6627 (18) Å between the ring centroids. In the crystal, C—H⋯O contacts link the mol­ecules into chains along the *b* axis.

## Related literature

For the synthesis and applications of the title compound, see: Yang & Lu (2010 ▶).
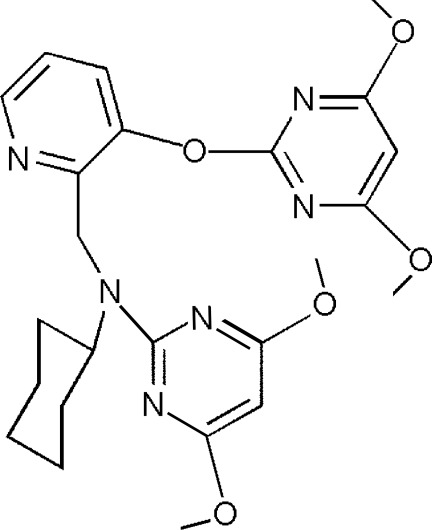


## Experimental

### 

#### Crystal data

C_24_H_30_N_6_O_5_
*M* *_r_* = 482.54
Triclinic, 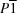

*a* = 7.0260 (14) Å
*b* = 10.624 (2) Å
*c* = 17.084 (3) Å
α = 72.95 (3)°
β = 84.18 (3)°
γ = 79.56 (3)°
*V* = 1197.4 (4) Å^3^
*Z* = 2
Mo *K*α radiation
μ = 0.10 mm^−1^
*T* = 293 K
0.30 × 0.20 × 0.10 mm

#### Data collection

Enraf–Nonius CAD-4 diffractometer
Absorption correction: multi-scan (*SADABS*; Sheldrick, 1996 ▶) *T*_min_ = 0.972, *T*_max_ = 0.991
4410 measured reflections
4410 independent reflections
2478 reflections with *I* > 2σ(*I*)
*R*_int_ = 0.000
3 standard reflections every 200 reflections intensity decay: 1%

#### Refinement

*R*[*F* ^2^ > 2σ(*F* ^2^)] = 0.063
*wR*(*F* ^2^) = 0.178
*S* = 1.00
4410 reflections
316 parameters
H-atom parameters constrained
Δρ_max_ = 0.22 e Å^−3^
Δρ_min_ = −0.21 e Å^−3^

### 

Data collection: *CAD-4 EXPRESS* (Enraf–Nonius, 1994 ▶); cell refinement: *CAD-4 EXPRESS*; data reduction: *XCAD4* (Harms & Wocadlo, 1995 ▶); program(s) used to solve structure: *SHELXS97* (Sheldrick, 2008 ▶); program(s) used to refine structure: *SHELXL97* (Sheldrick, 2008 ▶); molecular graphics: *SHELXTL* (Sheldrick, 2008 ▶); software used to prepare material for publication: *SHELXTL*.

## Supplementary Material

Crystal structure: contains datablock(s) I, global. DOI: 10.1107/S1600536812013104/sj5221sup1.cif

Structure factors: contains datablock(s) I. DOI: 10.1107/S1600536812013104/sj5221Isup2.hkl

Supplementary material file. DOI: 10.1107/S1600536812013104/sj5221Isup3.cml

Additional supplementary materials:  crystallographic information; 3D view; checkCIF report

## Figures and Tables

**Table 1:** Hydrogen-bond geometry (Å, °)

*D*—H⋯*A*	*D*—H	H⋯*A*	*D*⋯*A*	*D*—H⋯*A*
C18—H18*B*⋯O3^i^	0.96	2.69	3.477 (4)	140

Symmetry code: (i) 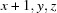.

**Fig. 1.**
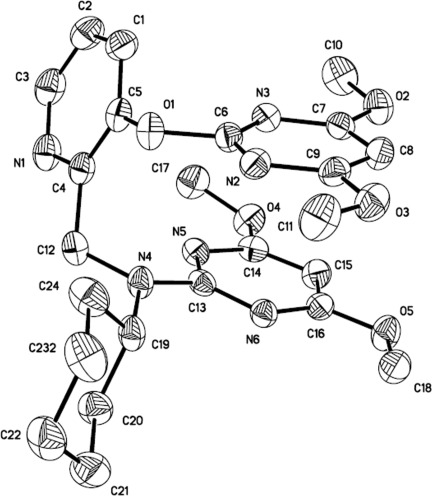
The structure of the title compound, showing the atomic numbering scheme. Non-H atoms are shown with 30% probability displacement ellipsoids.

## supplementary crystallographic information

### Comment

The title compound is an important organic intermediate for the synthesis of pyrimidine-oxy-*N*-aryl benzyl amine derivatives, important compounds for use as new pesticides Yang & Lu (2010). In the process of synthesizing one such derivative, we obtained crystals of the intermediate and we report its crystal structure herein.

As shown in Fig.1, the cyclohexyl ring(C19—C24) adopts a chair conformation, while the remainder of the molecule is U shaped. The dihedral angles between the central pyridyl ring(C1—C5/N1) and the pendant pyrimidine rings (C6—C9/N2/N3 and C13—C16/N5/N6) are 69.04 (12) and 75.99 (9)°, respectively. The the two pyrimidine rings are nearly parallel to each other, with a dihedral angle of 8.56 (15)° between them. An intramolecular π-π stacking interaction also occurs with a distance of 3.6627 (18)Å between the (C6—C9/N2/N3) and (C13—C16/N5/N6) ring centroids. In the crystal, molecules are linked by weak C18—H18B···O hydrogen-bonds forming chains along *b*.

### Experimental

The title compound was synthesized according to a published procedure (Yang & Lu, 2010). The product (0.3 g) was crystallized in methanol (15ml) at room temperature to give colorless crystals that were used for data collection.

### Refinement

All H atoms were placed in calculated positions and treated as riding: C—H = 0.93 and 0.96 Å for CH and CH_3_ H atoms, respectively, with *U*_iso_(H) = k × *U*_eq_(C), where k = 1.5 for CH_3_ H-atoms and k = 1.2 for all other H-atoms.

### Crystal data

**Table:**

C_24_H_30_N_6_O_5_	*Z* = 2
*M**_r_* = 482.54	*F*(000) = 512
Triclinic, *P*1	*D*_x_ = 1.338 Mg m^−^^3^
Hall symbol: -P 1	Mo *K*α radiation, λ = 0.71073 Å
*a* = 7.0260 (14) Å	Cell parameters from 25 reflections
*b* = 10.624 (2) Å	θ = 9–13°
*c* = 17.084 (3) Å	µ = 0.10 mm^−^^1^
α = 72.95 (3)°	*T* = 293 K
β = 84.18 (3)°	Block, white
γ = 79.56 (3)°	0.30 × 0.20 × 0.10 mm
*V* = 1197.4 (4) Å^3^	

### Data collection

**Table:**

Enraf–Nonius CAD-4 diffractometer	2478 reflections with *I* > 2σ(*I*)
Radiation source: fine-focus sealed tube	*R*_int_ = 0.000
Graphite monochromator	θ_max_ = 25.4°, θ_min_ = 1.3°
ω/2θ scans	*h* = −8→8
Absorption correction: multi-scan (*SADABS*; Sheldrick, 1996)	*k* = −12→12
*T*_min_ = 0.972, *T*_max_ = 0.991	*l* = 0→20
4410 measured reflections	3 standard reflections every 200 reflections
4410 independent reflections	intensity decay: 1%

### Refinement

**Table:**

Refinement on *F*^2^	Primary atom site location: structure-invariant direct methods
Least-squares matrix: full	Secondary atom site location: difference Fourier map
*R*[*F*^2^ > 2σ(*F*^2^)] = 0.063	Hydrogen site location: inferred from neighbouring sites
*wR*(*F*^2^) = 0.178	H-atom parameters constrained
*S* = 1.00	*w* = 1/[σ^2^(*F*_o_^2^) + (0.088*P*)^2^] where *P* = (*F*_o_^2^ + 2*F*_c_^2^)/3
4410 reflections	(Δ/σ)_max_ < 0.001
316 parameters	Δρ_max_ = 0.22 e Å^−^^3^
0 restraints	Δρ_min_ = −0.21 e Å^−^^3^

### Special details

**Table:**

Geometry. All e.s.d.'s (except the e.s.d. in the dihedral angle between two l.s. planes) are estimated using the full covariance matrix. The cell e.s.d.'s are taken into account individually in the estimation of e.s.d.'s in distances, angles and torsion angles; correlations between e.s.d.'s in cell parameters are only used when they are defined by crystal symmetry. An approximate (isotropic) treatment of cell e.s.d.'s is used for estimating e.s.d.'s involving l.s. planes.
Refinement. Refinement of *F*^2^ against ALL reflections. The weighted *R*-factor *wR* and goodness of fit *S* are based on *F*^2^, conventional *R*-factors *R* are based on *F*, with *F* set to zero for negative *F*^2^. The threshold expression of *F*^2^ > σ(*F*^2^) is used only for calculating *R*-factors(gt) *etc*. and is not relevant to the choice of reflections for refinement. *R*-factors based on *F*^2^ are statistically about twice as large as those based on *F*, and *R*- factors based on ALL data will be even larger.

### Fractional atomic coordinates and isotropic or equivalent isotropic displacement parameters (Å^2^)

**Table:**

	*x*	*y*	*z*	*U*_iso_*/*U*_eq_	
O1	−0.5779 (3)	−0.4010 (2)	0.30512 (12)	0.0540 (6)	
N1	−0.2211 (4)	−0.6995 (3)	0.32680 (17)	0.0611 (8)	
C1	−0.5799 (5)	−0.5959 (3)	0.2625 (2)	0.0603 (9)	
H1B	−0.7015	−0.5608	0.2416	0.072*	
N2	−0.6650 (3)	−0.1812 (2)	0.25672 (15)	0.0465 (6)	
N3	−0.5486 (4)	−0.3123 (2)	0.16500 (15)	0.0478 (6)	
O2	−0.5286 (3)	−0.2115 (2)	0.02694 (13)	0.0630 (6)	
C2	−0.4927 (6)	−0.7205 (4)	0.2608 (2)	0.0678 (10)	
H2B	−0.5505	−0.7711	0.2368	0.081*	
O3	−0.7485 (3)	0.0450 (2)	0.20679 (14)	0.0602 (6)	
C3	−0.3169 (6)	−0.7692 (3)	0.2957 (2)	0.0681 (10)	
H3A	−0.2613	−0.8564	0.2976	0.082*	
O4	0.0337 (3)	−0.4581 (2)	0.08476 (13)	0.0645 (7)	
N4	−0.1984 (4)	−0.3603 (2)	0.32456 (14)	0.0485 (7)	
C4	−0.3046 (5)	−0.5749 (3)	0.32542 (18)	0.0490 (8)	
O5	−0.1294 (3)	−0.0173 (2)	0.09128 (13)	0.0605 (6)	
N5	−0.0728 (3)	−0.4126 (2)	0.20658 (14)	0.0447 (6)	
C5	−0.4850 (5)	−0.5237 (3)	0.29541 (18)	0.0488 (8)	
N6	−0.1554 (3)	−0.1847 (2)	0.21032 (14)	0.0451 (6)	
C6	−0.5969 (4)	−0.2925 (3)	0.23752 (18)	0.0451 (7)	
C7	−0.5704 (4)	−0.2005 (3)	0.10297 (19)	0.0484 (8)	
C8	−0.6346 (5)	−0.0770 (3)	0.1132 (2)	0.0547 (8)	
H8A	−0.6451	−0.0001	0.0693	0.066*	
C9	−0.6827 (4)	−0.0735 (3)	0.1924 (2)	0.0486 (8)	
C10	−0.4772 (6)	−0.3424 (4)	0.0165 (2)	0.0772 (11)	
H10A	−0.4518	−0.3359	−0.0407	0.116*	
H10B	−0.5820	−0.3917	0.0375	0.116*	
H10C	−0.3633	−0.3875	0.0458	0.116*	
C11	−0.8112 (6)	0.0430 (3)	0.2890 (2)	0.0740 (11)	
H11A	−0.8558	0.1326	0.2918	0.111*	
H11B	−0.7050	0.0034	0.3241	0.111*	
H11C	−0.9149	−0.0083	0.3065	0.111*	
C12	−0.1847 (5)	−0.5041 (3)	0.36202 (19)	0.0537 (8)	
H12A	−0.2232	−0.5194	0.4198	0.064*	
H12B	−0.0500	−0.5445	0.3583	0.064*	
C13	−0.1381 (4)	−0.3174 (3)	0.24449 (17)	0.0421 (7)	
C14	−0.0298 (4)	−0.3675 (3)	0.12722 (19)	0.0483 (8)	
C15	−0.0444 (5)	−0.2355 (3)	0.08470 (19)	0.0536 (8)	
H15A	−0.0130	−0.2069	0.0288	0.064*	
C16	−0.1090 (4)	−0.1485 (3)	0.13068 (18)	0.0462 (8)	
C17	0.0071 (6)	−0.5926 (4)	0.1224 (2)	0.0745 (11)	
H17A	0.0587	−0.6458	0.0860	0.112*	
H17B	−0.1286	−0.5964	0.1338	0.112*	
H17C	0.0734	−0.6263	0.1726	0.112*	
C18	−0.2060 (5)	0.0734 (3)	0.1364 (2)	0.0599 (9)	
H18A	−0.2129	0.1628	0.1012	0.090*	
H18B	−0.1239	0.0615	0.1805	0.090*	
H18C	−0.3336	0.0577	0.1584	0.090*	
C19	−0.2291 (5)	−0.2710 (3)	0.37820 (17)	0.0505 (8)	
H19A	−0.2527	−0.1798	0.3419	0.061*	
C20	−0.0472 (5)	−0.2818 (3)	0.4213 (2)	0.0613 (9)	
H20A	0.0598	−0.2649	0.3811	0.074*	
H20B	−0.0156	−0.3715	0.4572	0.074*	
C21	−0.0747 (6)	−0.1815 (4)	0.4717 (2)	0.0773 (12)	
H21A	0.0391	−0.1959	0.5028	0.093*	
H21B	−0.0871	−0.0918	0.4348	0.093*	
C22	−0.2499 (6)	−0.1934 (4)	0.5293 (2)	0.0775 (12)	
H22A	−0.2686	−0.1229	0.5560	0.093*	
H22B	−0.2298	−0.2782	0.5713	0.093*	
C232	−0.4279 (6)	−0.1842 (4)	0.4849 (2)	0.0811 (12)	
H23A	−0.4566	−0.0956	0.4475	0.097*	
H23B	−0.5371	−0.1980	0.5243	0.097*	
C24	−0.4025 (5)	−0.2862 (4)	0.4373 (2)	0.0696 (11)	
H24A	−0.3858	−0.3752	0.4750	0.084*	
H24B	−0.5176	−0.2747	0.4075	0.084*	

### Atomic displacement parameters (Å^2^)

**Table:**

	*U*^11^	*U*^22^	*U*^33^	*U*^12^	*U*^13^	*U*^23^
O1	0.0638 (15)	0.0462 (13)	0.0439 (12)	0.0004 (10)	0.0010 (11)	−0.0068 (10)
N1	0.078 (2)	0.0425 (17)	0.0560 (17)	0.0019 (14)	0.0017 (15)	−0.0122 (13)
C1	0.058 (2)	0.059 (2)	0.065 (2)	−0.0150 (18)	−0.0010 (18)	−0.0151 (18)
N2	0.0416 (15)	0.0436 (15)	0.0524 (16)	−0.0058 (12)	−0.0014 (12)	−0.0117 (13)
N3	0.0482 (16)	0.0485 (16)	0.0466 (15)	−0.0090 (12)	−0.0040 (12)	−0.0117 (13)
O2	0.0733 (17)	0.0669 (16)	0.0448 (13)	−0.0085 (12)	−0.0014 (11)	−0.0116 (11)
C2	0.079 (3)	0.054 (2)	0.075 (3)	−0.018 (2)	0.004 (2)	−0.0232 (19)
O3	0.0629 (15)	0.0441 (14)	0.0702 (16)	−0.0059 (11)	0.0003 (12)	−0.0137 (11)
C3	0.093 (3)	0.041 (2)	0.069 (2)	−0.007 (2)	0.004 (2)	−0.0182 (18)
O4	0.0783 (17)	0.0678 (16)	0.0531 (14)	−0.0184 (13)	0.0168 (12)	−0.0282 (12)
N4	0.0631 (18)	0.0460 (15)	0.0341 (13)	−0.0075 (12)	0.0016 (12)	−0.0098 (11)
C4	0.060 (2)	0.0434 (19)	0.0406 (17)	−0.0065 (16)	0.0054 (15)	−0.0100 (14)
O5	0.0741 (16)	0.0578 (15)	0.0450 (12)	−0.0160 (12)	0.0096 (12)	−0.0080 (11)
N5	0.0443 (15)	0.0508 (16)	0.0396 (14)	−0.0043 (12)	−0.0007 (11)	−0.0159 (12)
C5	0.058 (2)	0.0387 (18)	0.0433 (17)	−0.0031 (15)	0.0043 (16)	−0.0072 (14)
N6	0.0442 (16)	0.0504 (16)	0.0394 (14)	−0.0098 (12)	−0.0010 (12)	−0.0097 (12)
C6	0.0408 (18)	0.0456 (19)	0.0443 (18)	−0.0062 (14)	−0.0032 (14)	−0.0057 (14)
C7	0.0439 (19)	0.057 (2)	0.0427 (18)	−0.0137 (15)	−0.0047 (14)	−0.0083 (16)
C8	0.055 (2)	0.047 (2)	0.056 (2)	−0.0108 (16)	−0.0042 (17)	−0.0024 (16)
C9	0.0404 (18)	0.0412 (19)	0.064 (2)	−0.0068 (14)	−0.0050 (16)	−0.0129 (16)
C10	0.084 (3)	0.087 (3)	0.061 (2)	−0.001 (2)	0.001 (2)	−0.032 (2)
C11	0.086 (3)	0.054 (2)	0.080 (3)	−0.0045 (19)	0.009 (2)	−0.024 (2)
C12	0.064 (2)	0.050 (2)	0.0414 (17)	−0.0009 (16)	−0.0050 (16)	−0.0083 (15)
C13	0.0374 (17)	0.056 (2)	0.0339 (16)	−0.0076 (14)	−0.0015 (13)	−0.0146 (14)
C14	0.0407 (19)	0.065 (2)	0.0447 (18)	−0.0107 (15)	0.0062 (14)	−0.0256 (17)
C15	0.060 (2)	0.064 (2)	0.0394 (17)	−0.0178 (17)	0.0090 (16)	−0.0170 (16)
C16	0.0400 (18)	0.054 (2)	0.0431 (18)	−0.0133 (15)	0.0021 (14)	−0.0096 (15)
C17	0.096 (3)	0.063 (3)	0.071 (3)	−0.014 (2)	0.005 (2)	−0.032 (2)
C18	0.069 (2)	0.055 (2)	0.056 (2)	−0.0132 (17)	−0.0010 (18)	−0.0146 (17)
C19	0.069 (2)	0.0458 (19)	0.0331 (16)	−0.0043 (16)	0.0013 (15)	−0.0098 (14)
C20	0.065 (2)	0.070 (2)	0.053 (2)	−0.0135 (18)	0.0004 (18)	−0.0237 (18)
C21	0.099 (3)	0.084 (3)	0.063 (2)	−0.029 (2)	−0.001 (2)	−0.035 (2)
C22	0.108 (3)	0.080 (3)	0.051 (2)	−0.010 (2)	−0.001 (2)	−0.032 (2)
C232	0.088 (3)	0.099 (3)	0.055 (2)	0.008 (2)	0.005 (2)	−0.036 (2)
C24	0.062 (2)	0.097 (3)	0.054 (2)	−0.007 (2)	0.0021 (18)	−0.033 (2)

### Geometric parameters (Å, º)

**Table:**

O1—C6	1.370 (3)	C10—H10A	0.9600
O1—C5	1.396 (3)	C10—H10B	0.9600
N1—C3	1.331 (4)	C10—H10C	0.9600
N1—C4	1.342 (4)	C11—H11A	0.9600
C1—C2	1.361 (4)	C11—H11B	0.9600
C1—C5	1.366 (4)	C11—H11C	0.9600
C1—H1B	0.9300	C12—H12A	0.9700
N2—C6	1.314 (4)	C12—H12B	0.9700
N2—C9	1.331 (4)	C14—C15	1.368 (4)
N3—C6	1.318 (4)	C15—C16	1.371 (4)
N3—C7	1.337 (4)	C15—H15A	0.9300
O2—C7	1.337 (4)	C17—H17A	0.9600
O2—C10	1.431 (4)	C17—H17B	0.9600
C2—C3	1.373 (5)	C17—H17C	0.9600
C2—H2B	0.9300	C18—H18A	0.9600
O3—C9	1.344 (3)	C18—H18B	0.9600
O3—C11	1.424 (4)	C18—H18C	0.9600
C3—H3A	0.9300	C19—C24	1.502 (4)
O4—C14	1.357 (3)	C19—C20	1.509 (4)
O4—C17	1.422 (4)	C19—H19A	0.9800
N4—C13	1.358 (4)	C20—C21	1.531 (4)
N4—C12	1.462 (4)	C20—H20A	0.9700
N4—C19	1.478 (4)	C20—H20B	0.9700
C4—C5	1.372 (4)	C21—C22	1.496 (5)
C4—C12	1.509 (4)	C21—H21A	0.9700
O5—C16	1.346 (3)	C21—H21B	0.9700
O5—C18	1.405 (4)	C22—C232	1.501 (5)
N5—C14	1.322 (4)	C22—H22A	0.9700
N5—C13	1.344 (4)	C22—H22B	0.9700
N6—C16	1.324 (4)	C232—C24	1.511 (5)
N6—C13	1.347 (4)	C232—H23A	0.9700
C7—C8	1.363 (4)	C232—H23B	0.9700
C8—C9	1.370 (4)	C24—H24A	0.9700
C8—H8A	0.9300	C24—H24B	0.9700
			
C6—O1—C5	118.7 (2)	N5—C13—N4	116.2 (3)
C3—N1—C4	117.5 (3)	N6—C13—N4	117.2 (3)
C2—C1—C5	118.6 (3)	N5—C14—O4	117.9 (3)
C2—C1—H1B	120.7	N5—C14—C15	124.6 (3)
C5—C1—H1B	120.7	O4—C14—C15	117.5 (3)
C6—N2—C9	113.8 (3)	C14—C15—C16	115.0 (3)
C6—N3—C7	113.6 (3)	C14—C15—H15A	122.5
C7—O2—C10	118.1 (3)	C16—C15—H15A	122.5
C1—C2—C3	118.0 (3)	N6—C16—O5	118.2 (3)
C1—C2—H2B	121.0	N6—C16—C15	124.5 (3)
C3—C2—H2B	121.0	O5—C16—C15	117.2 (3)
C9—O3—C11	116.9 (3)	O4—C17—H17A	109.5
N1—C3—C2	124.1 (3)	O4—C17—H17B	109.5
N1—C3—H3A	118.0	H17A—C17—H17B	109.5
C2—C3—H3A	118.0	O4—C17—H17C	109.5
C14—O4—C17	118.1 (3)	H17A—C17—H17C	109.5
C13—N4—C12	117.9 (2)	H17B—C17—H17C	109.5
C13—N4—C19	121.4 (2)	O5—C18—H18A	109.5
C12—N4—C19	118.9 (2)	O5—C18—H18B	109.5
N1—C4—C5	120.9 (3)	H18A—C18—H18B	109.5
N1—C4—C12	113.4 (3)	O5—C18—H18C	109.5
C5—C4—C12	125.7 (3)	H18A—C18—H18C	109.5
C16—O5—C18	118.1 (2)	H18B—C18—H18C	109.5
C14—N5—C13	114.8 (3)	N4—C19—C24	114.9 (3)
C1—C5—C4	120.8 (3)	N4—C19—C20	110.8 (3)
C1—C5—O1	119.9 (3)	C24—C19—C20	111.6 (3)
C4—C5—O1	119.0 (3)	N4—C19—H19A	106.3
C16—N6—C13	114.6 (3)	C24—C19—H19A	106.3
N2—C6—N3	129.7 (3)	C20—C19—H19A	106.3
N2—C6—O1	112.3 (3)	C19—C20—C21	110.6 (3)
N3—C6—O1	118.0 (3)	C19—C20—H20A	109.5
O2—C7—N3	117.7 (3)	C21—C20—H20A	109.5
O2—C7—C8	118.7 (3)	C19—C20—H20B	109.5
N3—C7—C8	123.6 (3)	C21—C20—H20B	109.5
C7—C8—C9	115.6 (3)	H20A—C20—H20B	108.1
C7—C8—H8A	122.2	C22—C21—C20	112.2 (3)
C9—C8—H8A	122.2	C22—C21—H21A	109.2
N2—C9—O3	117.6 (3)	C20—C21—H21A	109.2
N2—C9—C8	123.7 (3)	C22—C21—H21B	109.2
O3—C9—C8	118.7 (3)	C20—C21—H21B	109.2
O2—C10—H10A	109.5	H21A—C21—H21B	107.9
O2—C10—H10B	109.5	C21—C22—C232	111.3 (3)
H10A—C10—H10B	109.5	C21—C22—H22A	109.4
O2—C10—H10C	109.5	C232—C22—H22A	109.4
H10A—C10—H10C	109.5	C21—C22—H22B	109.4
H10B—C10—H10C	109.5	C232—C22—H22B	109.4
O3—C11—H11A	109.5	H22A—C22—H22B	108.0
O3—C11—H11B	109.5	C22—C232—C24	111.8 (3)
H11A—C11—H11B	109.5	C22—C232—H23A	109.3
O3—C11—H11C	109.5	C24—C232—H23A	109.3
H11A—C11—H11C	109.5	C22—C232—H23B	109.3
H11B—C11—H11C	109.5	C24—C232—H23B	109.3
N4—C12—C4	115.9 (3)	H23A—C232—H23B	107.9
N4—C12—H12A	108.3	C19—C24—C232	110.5 (3)
C4—C12—H12A	108.3	C19—C24—H24A	109.6
N4—C12—H12B	108.3	C232—C24—H24A	109.6
C4—C12—H12B	108.3	C19—C24—H24B	109.6
H12A—C12—H12B	107.4	C232—C24—H24B	109.6
N5—C13—N6	126.5 (3)	H24A—C24—H24B	108.1
			
C5—C1—C2—C3	2.7 (5)	C5—C4—C12—N4	−35.9 (4)
C4—N1—C3—C2	1.5 (5)	C14—N5—C13—N6	−2.8 (4)
C1—C2—C3—N1	−4.0 (6)	C14—N5—C13—N4	174.9 (3)
C3—N1—C4—C5	2.1 (4)	C16—N6—C13—N5	2.8 (4)
C3—N1—C4—C12	−179.9 (3)	C16—N6—C13—N4	−174.9 (3)
C2—C1—C5—C4	0.7 (5)	C12—N4—C13—N5	0.8 (4)
C2—C1—C5—O1	−173.9 (3)	C19—N4—C13—N5	165.3 (3)
N1—C4—C5—C1	−3.3 (5)	C12—N4—C13—N6	178.6 (2)
C12—C4—C5—C1	179.1 (3)	C19—N4—C13—N6	−16.8 (4)
N1—C4—C5—O1	171.4 (3)	C13—N5—C14—O4	−178.8 (2)
C12—C4—C5—O1	−6.2 (5)	C13—N5—C14—C15	1.3 (4)
C6—O1—C5—C1	−76.2 (4)	C17—O4—C14—N5	14.6 (4)
C6—O1—C5—C4	109.0 (3)	C17—O4—C14—C15	−165.5 (3)
C9—N2—C6—N3	−1.5 (5)	N5—C14—C15—C16	0.0 (5)
C9—N2—C6—O1	179.2 (2)	O4—C14—C15—C16	−179.9 (3)
C7—N3—C6—N2	1.3 (5)	C13—N6—C16—O5	177.5 (2)
C7—N3—C6—O1	−179.4 (3)	C13—N6—C16—C15	−1.2 (4)
C5—O1—C6—N2	−172.2 (3)	C18—O5—C16—N6	−2.4 (4)
C5—O1—C6—N3	8.4 (4)	C18—O5—C16—C15	176.4 (3)
C10—O2—C7—N3	4.5 (4)	C14—C15—C16—N6	0.0 (5)
C10—O2—C7—C8	−174.8 (3)	C14—C15—C16—O5	−178.8 (3)
C6—N3—C7—O2	−178.6 (3)	C13—N4—C19—C24	140.8 (3)
C6—N3—C7—C8	0.6 (4)	C12—N4—C19—C24	−54.8 (4)
O2—C7—C8—C9	177.3 (3)	C13—N4—C19—C20	−91.5 (3)
N3—C7—C8—C9	−2.0 (5)	C12—N4—C19—C20	72.9 (3)
C6—N2—C9—O3	−179.0 (2)	N4—C19—C20—C21	175.7 (3)
C6—N2—C9—C8	−0.1 (4)	C24—C19—C20—C21	−54.9 (4)
C11—O3—C9—N2	−5.9 (4)	C19—C20—C21—C22	53.5 (4)
C11—O3—C9—C8	175.2 (3)	C20—C21—C22—C232	−53.7 (5)
C7—C8—C9—N2	1.7 (5)	C21—C22—C232—C24	55.3 (5)
C7—C8—C9—O3	−179.5 (3)	N4—C19—C24—C232	−176.2 (3)
C13—N4—C12—C4	−62.4 (4)	C20—C19—C24—C232	56.6 (4)
C19—N4—C12—C4	132.7 (3)	C22—C232—C24—C19	−56.5 (4)
N1—C4—C12—N4	146.3 (3)		

### Hydrogen-bond geometry (Å, º)

**Table:**

*D*—H···*A*	*D*—H	H···*A*	*D*···*A*	*D*—H···*A*
C18—H18*B*···O3^i^	0.96	2.69	3.477 (4)	140

Symmetry code: (i) *x*+1, *y*, *z*.
